# The “Jokari Sign”, An Imaging Feature Diagnostic of a Wandering Accessory Spleen

**DOI:** 10.5334/jbr-btr.857

**Published:** 2015-09-15

**Authors:** N. Vander Maren, N. Verbeeck

**Affiliations:** 1 Cliniques Universitaires Saint-Luc, 10, avenue Hippocrate, B-1200 Bruxelles, Belgium; 2 Department of Radiology, Centre Hospitalier de Luxembourg, Luxembourg, Grand Duchy of Luxembourg

**Keywords:** Spleen, abnormalities

## Abstract

If cross-sectional imaging techniques often disclose the presence of an accessory spleen, they seldom detect a wandering accessory spleen. This latter diagnosis can be challenging but important and derives great benefit from computed tomography with curved multiplanar and three-dimensional reconstructions displaying the long vascular pedicle connecting the small mass to the splenic vessels. We call this anatomical complex the “Jokari sign”, in reference to the ball-on-a-string racket game.

The diagnosis of a wandering accessory spleen (WAS) may prove delicate. It is nevertheless most important in three clinical scenarios. First, the nodule can mimic a lymphadenopathy or an abdominal tumor, second, the WAS can occasionally trigger symptoms in case of torsion, rupture or hemorrhage, third, its existence must be recognized when the functional splenic tissue must be surgically resected.

A WAS will essentially be identified on the basis of cross-sectional imaging techniques. The excellent spatial resolution of computed tomography (CT), in particular, enables a correct diagnosis based essentially on curved multiplanar and three-dimensional (3D) reconstructions.

## Case report

**Figure 1:**
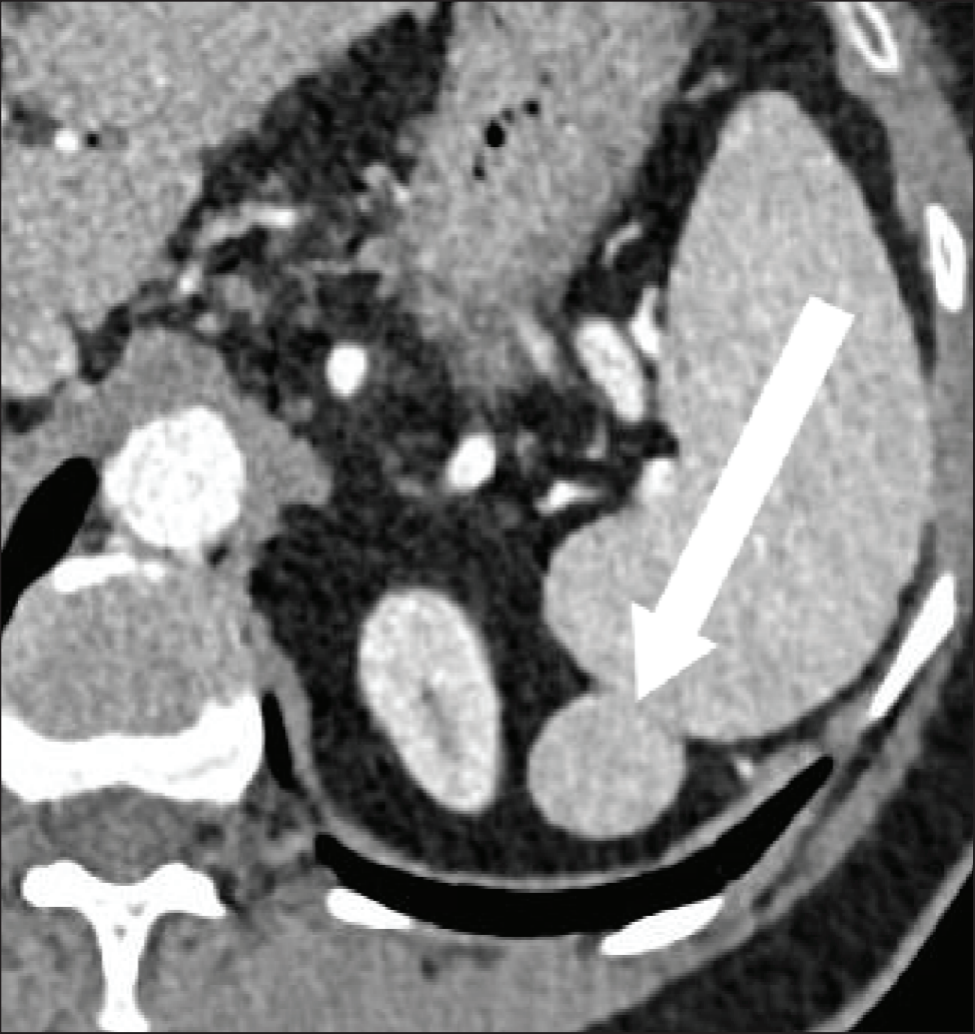
Axial enhanced CT slice: the WAS (arrow) between the upper renal pole and the spleen.

A 60-year old woman with a right breast neoplasm is presented to the Radiology Department for a thoraco-abdominal CT scan. The only anomaly displayed on the axial images is a well-marginated, 3 cm in diameter, enhancing mass at the level of the left upper abdominal quadrant between the spleen and the diaphragm. Although the lesion resembles an accessory spleen based on similar enhancement characteristics of the main spleen, a metastatic nodule is not entirely excluded (Fig. 1).

**Figure 2:**
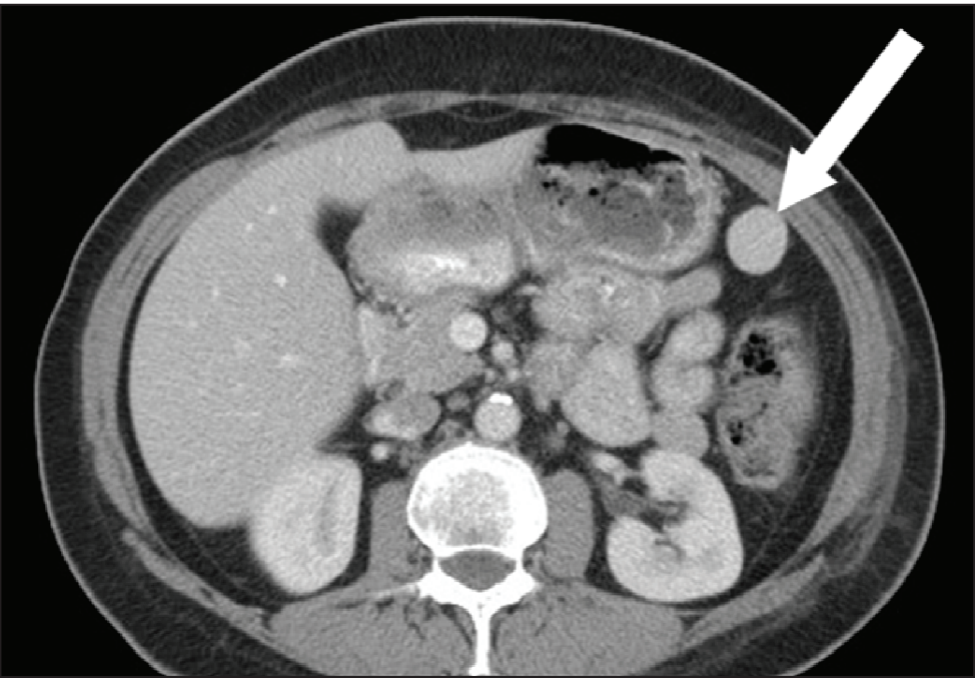
Three years ago, a more caudal slice of the same patient as in Fig. 1: the WAS between the stomach and the left colon.

Since the patient underwent previous thoracic CT scans for recurrent pneumonia, we reviewed those displaying some upper abdominal axial images. The nodule was already visible but its location was different: it was then spotted anterior to the lower pole of the spleen, between the greater curvature of the stomach and the upper left colon (Fig. 2).

**Figure 3:**
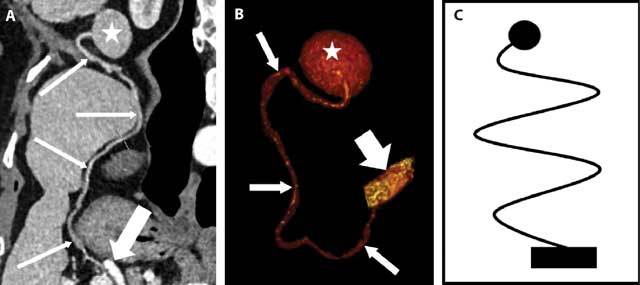
Curved multipanar (A) and 3D (B) CT reconstructions besides a schematic drawing of the “Jokari sign”: the WAS (star), its long, thin vascular pedicle (thin arrows) and the splenic artery (thick arrow).

An examination of the thin slices of the last oncological CT reveals that the small mass is fed by an 18 cm-long thin vascular pedicle that we submit to curved multiplanar and 3D reconstructions. The obvious link between the nodule and the splenic vessels, a complex that we call the “Jokari sign”, enables us to give a correct diagnosis of a WAS (Figs. 3A–C).

## Discussion

The spleen is an intra-peritoneal lymphoid organ weighing about 200 g usually located in the left upper abdominal quadrant under the rib cage. It is maintained in its position by two ligaments: the gastrosplenic ligament, which links the greater curvature of the stomach to the ventral face of the spleen, and the splenorenal ligament extending between the left kidney and the spleen, attaching the latter to the posterior abdominal wall. The spleen is more or less a tetrahedron whose shape is conditioned by the neighboring organs, among others, the diaphragm and the left kidney. Though it is most often unique and homogeneous, the spleen may display notches, even clefts, which are remnants of the fetal lobulation of the organ. These variants must be recognized in order not to be mistaken for expansive loco-regional lesions [1].

**Figure 4A–C:**
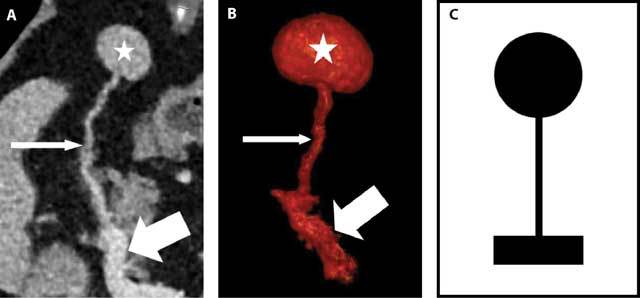
Curved multiplanar (A) and 3D (B) CT reconstructions besides a schematic drawing of the “beauty mirror sign” (C): the accessory spleen (star), its short vascular pedicle (thin arrow) and the splenic artery (thick arrow).

Accessory spleens result from a failure of fusion of the splenic buds, during the fifth week of fetal life, in the dorsal mesogastrium [2]. According to the literature, accessory spleens are seen in 10 to 30 % of the population [3, 4]. They are most often located near the splenic hilum (60%) and the inferior pole of the spleen (33%) but other sites are possible [5]. Their size may vary from a few millimeters to several centimeters and their diameter rarely exceeds 4 cm whereas their number ranges between 1 and 6 by patient [1, 4]. The diagnosis of accessory spleens is important in three clinical situations. First, the accessory spleen can mimic an adenopathy or an abdominal tumor with a possible pancreatic, suprarenal or renal origin. Second, an accessory spleen can occasionally become symptomatic in case of torsion, rupture or hemorrhage. Third, the surgeon must be warned of the existence of one or more accessory spleens when a total splenectomy is recommended like in some cases of hematologic disorders [2]. The diagnosis of an accessory spleen derives great benefit from cross-sectional imaging techniques and particularly from CT, whose spatial resolution is exceptional [3, 6]. A non-complicated accessory spleen enhances, after injection of intravenous contrast material, just like the normal splenic parenchyma whereas curved multiplanar and 3D reconstructions enable the identification of the short vascular pedicle which joins the small mass to the splenic vessels: we call this presentation the “beauty mirror sign” [Fig. 4A–C]. Let us not forget that, in case of splenosis, which results from the abdominal dissemination of splenic islets after a surgery or a trauma of the spleen, the nodule enhances after intravenous contrast administration, similarly to the splenic parenchyma but without any possibility to demonstrate a “beauty mirror sign”.

The wandering spleen is a rare entity whose incidence is estimated at about 1.5% and where the organ is found outside its usual location. The etiology of this presentation results from a laxity or a maldevelopment of the splenic ligaments with, as a corollary, a ubiquitous splenic positioning in the abdomino-pelvic area depending on the length of the vascular pedicle [4, 7]. Most frequently, a wandering spleen remains asymptomatic and is thus discovered incidentally. Ultrasonography, preferably used with children, but mainly CT, even magnetic resonance, enable a diagnosis. Signs denoting a wandering spleen are the absence of a spleen in its normal anatomic position and the presence of a mass, located somewhere in the abdomino-pelvic cavity, displaying a similar enhancement, after intravenous opacification, to that of a normal splenic parenchyma. A wandering spleen can cause abdominal pains in case of torsion or rupture. In case of torsion, the whirl sign of the vessels and the partial or complete lack of enhancement of the mass enable to make the diagnosis.

The WAS remains an extremely rare occurrence with only few cases described in the literature and in which an accessory spleen with a long vascular pedicle is mobile in the abdomino-pelvic cavity [1, 2, 8]. The differential diagnosis of the WAS rests mainly on the contrast enhanced CT with curved multiplanar and 3D reconstructions that demonstrate a long, thin vascular pedicle linking the mass to the splenic vessels or “Jokari sign”. The WAS generally remains asymptomatic but it can trigger acute abdominal pains in case of torsion, rupture or hemorrhage. In case of ischemic or hemorrhagic complication of a WAS, the “Jokari sign” remains of course present but the density of the “ball”, hypodense after contrast or spontaneously hyperdense respectively, differs from that of the normal splenic parenchyma.

## Conclusion

A WAS is an extremely rare entity which must nevertheless be recognized in the frame of oncological evaluations, during painful abdominal episodes or when a hemato-oncological surgery has been programmed.

Its correct diagnosis is essentially based on CT, whose incomparable spatial resolution enables multiplanar and 3D reconstructions of the long vascular pedicle connecting the small mass to the splenic vessels or “Jokari sign”.

## Competing Interests

The authors declare that they have no competing interests.
